# Supplementary material for: Anthropomorphic Characterization of Ankle Joint
Source: Bioengineering (Basel). 2023 Oct 17;10(10):1212. doi: 10.3390/bioengineering10101212 (PMC10604250; doi:10.3390/bioengineering10101212)
Supplement: Supplementary file 1 [file bioengineering-10-01212-s001.zip › bioengineering-2587353-supplementary.pdf]

Supplementary Data

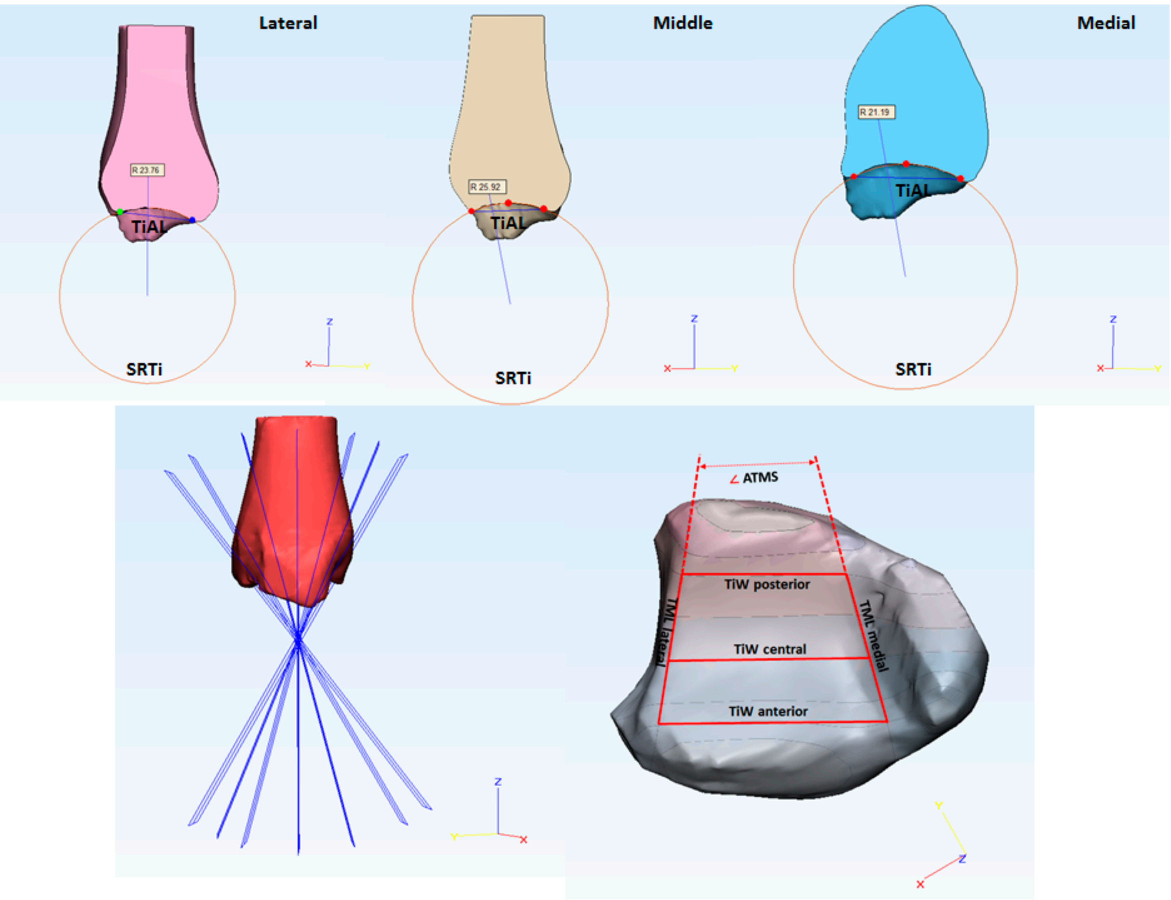

Figure S1. Measurement of morphological parameters of the tibia in sagittal and coronal planes.

Table S1. P-values obtained for the difference between talar edge angles and between radius values. Values that are significant are marked with an asterisk (*).

**Talar edge angle (deg)**						
	**α anterior**	**β anterior**	**α central**	**β central**	**α posterior**	**β posterior**
**α anterior**	-	0.844	0.001*	0.130	0.263	0.339
**β anterior**	0.844	-	0.004*	0.253	0.363	0.253
**α central**	0.001*	0.004*	-	0.014*	<0.0001*	0.011*
**β central**	0.130	0.253	0.014*	-	0.017*	0.747
**α posterior**	0.263	0.363	<0.0001*	0.017*	-	0.032*
**β posterior**	0.339	0.253	0.011*	0.747	0.032*	-
**Talar edge radius (mm)**						
	**R_l anterior**	**R_m anterior**	**R_l central**	**R_m central**	**R_l posterior**	**R_m posterior**
**R_l anterior**	-	0.020*	0.466	0.018*	0.006*	0.037*
**R_m anterior**	0.020*	-	0.088	0.126	0.043*	0.950
**R_l central**	0.466	0.088	-	0.004*	0.003*	0.037*
**R_m central**	0.018*	0.126	0.004*	-	0.677	0.109
**R_l posterior**	0.006*	0.043*	0.003*	0.677	-	0.024
**R_m posterior**	0.037*	0.950	0.037*	0.109	0.024	-

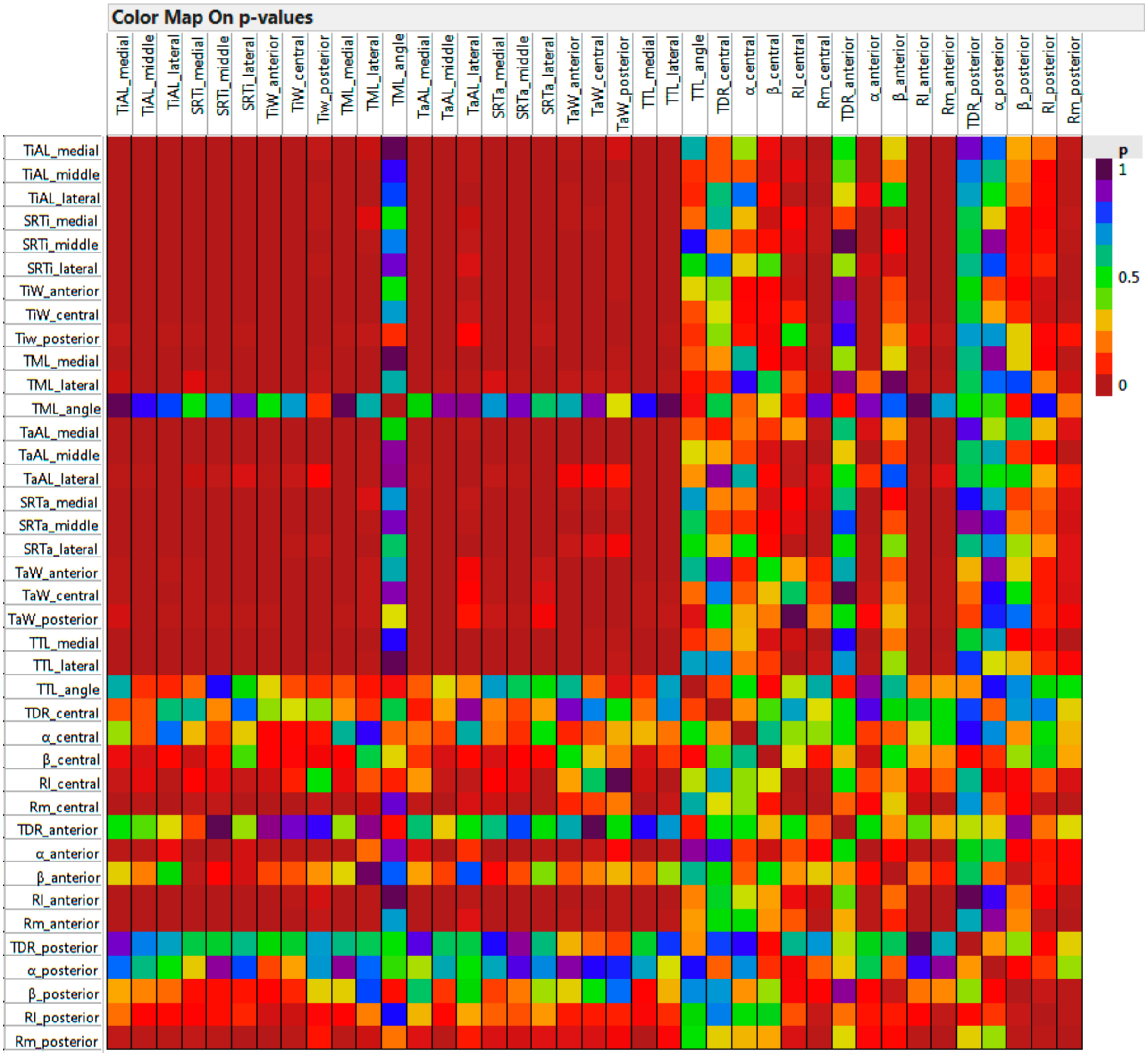

Figure S2. The significance of correlation between morphological parameters (p-values) obtained for tibia and talus (p=0 (red, <0.05) – evidence that significant correlation exists between variables and p=1 (blue) – no evidence that significant correlation exists between variables).

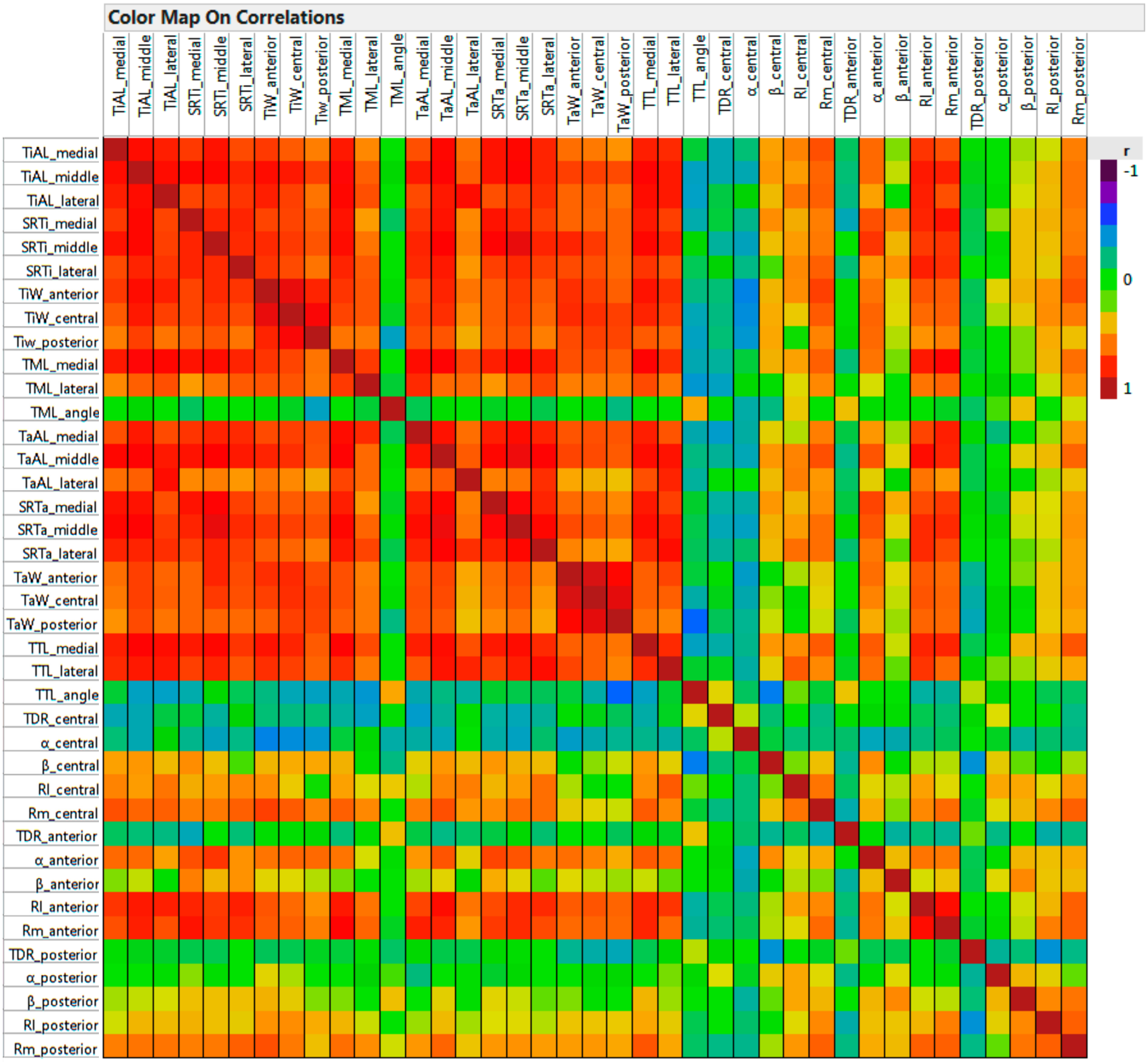

Figure S3. Correlations between morphological parameters (r-values) obtained for tibia and talus (r = 1 (red) – positive correlation exists between the variables, r = 0 (green) – no correlation exists between the variables and r = -1 (blue) – negative correlation exists between the variables).

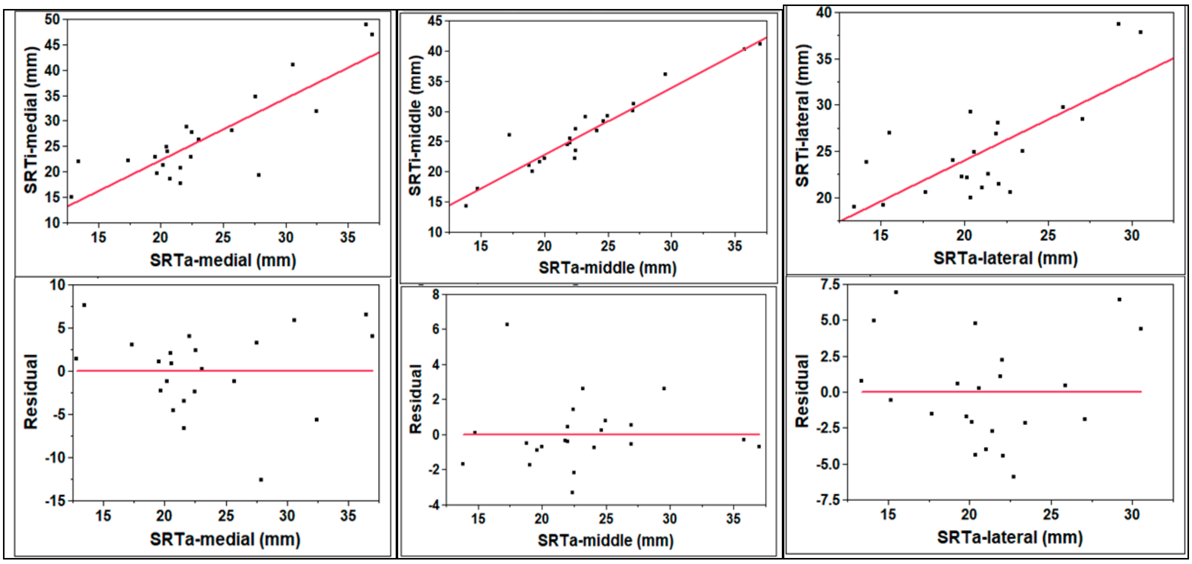

Figure S4. Regression plot (top row) and respective Residuals versus Fitted values plot (bottom row) for talus sagittal radius values (response is tibia sagittal radius) in different sections (medial, middle and lateral).

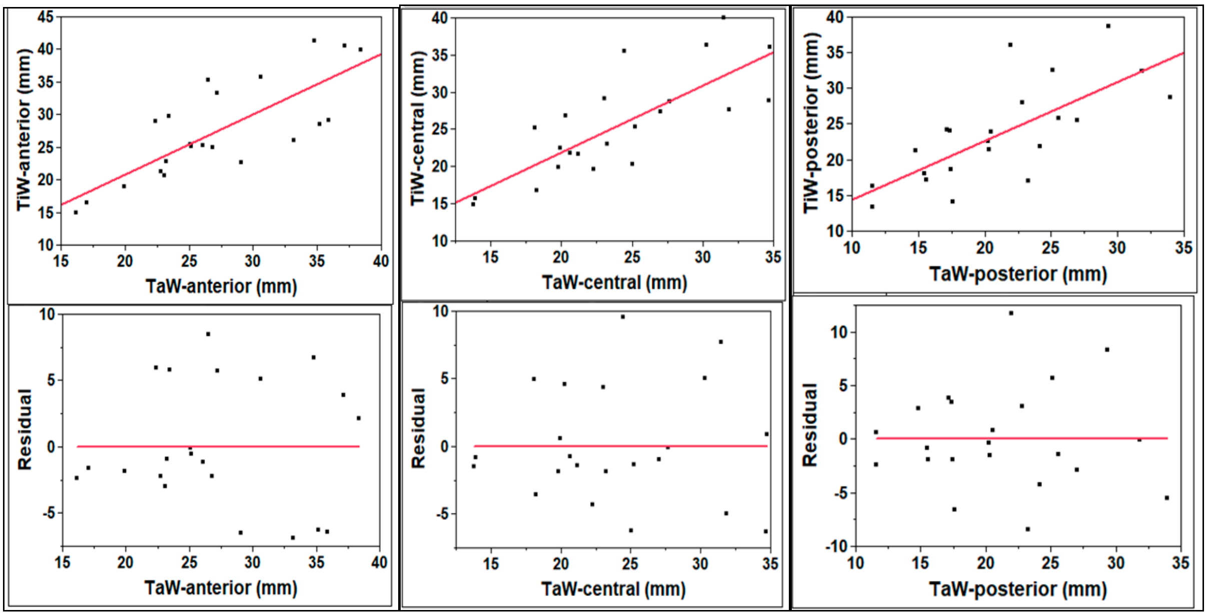

Figure S5. Regression plot (top row) and respective Residuals versus Fitted values plot (bottom row) for talus width values (response is tibia width radius) in different sections (anterior, central and posterior).

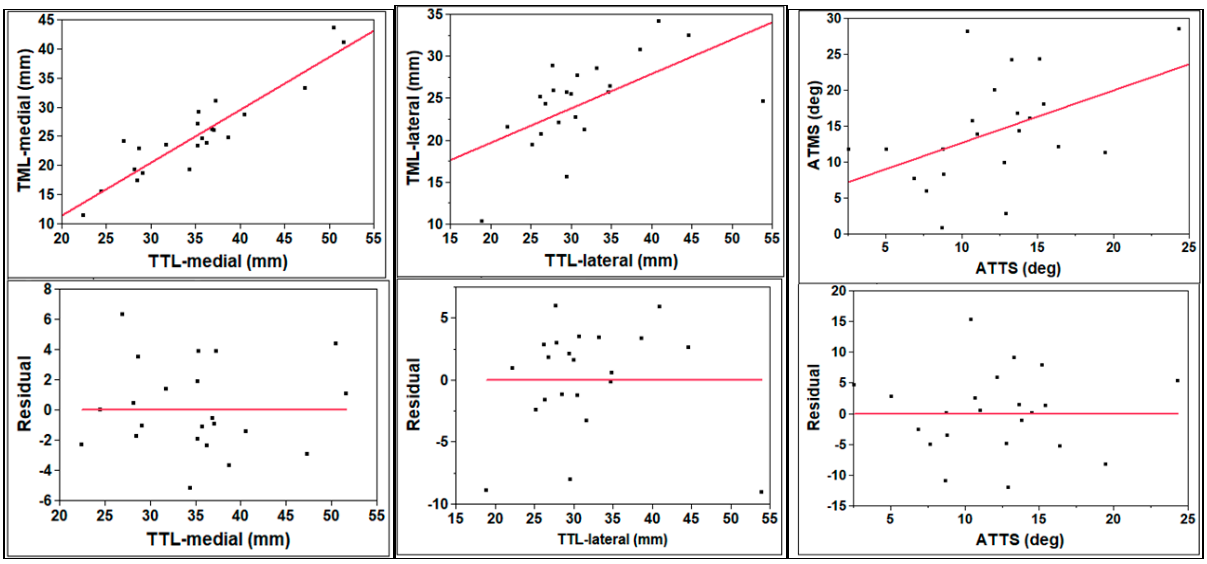

Figure S6. Regression plot (top row) and respective Residuals versus Fitted values plot (bottom row) for trochlea tali length and angle values (response is tibial mortise length and angle values) in different sections (medial, lateral), respectively.
